# Environmental and Economic Impacts of Substituting Single-Use Plastic Straws: A Life-Cycle Assessment for Greece

**DOI:** 10.3390/polym17091235

**Published:** 2025-04-30

**Authors:** Panagiota Eleni, Christos Boukouvalas

**Affiliations:** 1 Exelisis IKE, Consulting Company, Leof. Dekelias 215 & Skra 2, 14342 Athens, Greece; 2 School of Chemical Engineering, National Technical University of Athens, Iroon Polytechneiou 9, Zografou Campus, 15780 Athens, Greece; cbouk@chemeng.ntua.gr

**Keywords:** consumer preferences, environmental impact, Greece, life-cycle assessment (LCA), plastic straw alternatives, reusable straws, single-use plastics, sustainability

## Abstract

The usage of more than 30 billion straws a year has been reported in the European Union (EU), in 2020, one year before the official ban of single-use plastics in Europe. The impacts of this plastic waste on the environment and on our health are global and can be drastic. Since then, various alternative straws have emerged. This study assesses their effectiveness, primarily from an environmental perspective, to determine the best option among those available. Life-cycle assessment (LCA) was conducted using ReCiPe 2016 methodology and ISO 14040/44 standards, alongside a preliminary cost analysis and a consumer preference survey. The findings reveal that wheat straws demonstrated the lowest overall environmental impact, with a climate change contribution of only 0.0568 kg CO_2_ eq. per year, while plastic straws showed the lowest cost at EUR 0.30 per year but contributed 0.084 kg CO_2_ eq. Metallic straws, despite being reusable, had the highest washing-related emissions, with 85% of their annual impact (~0.169 kg CO_2_ eq.) attributed to dishwashing. Paper and bioplastic alternatives showed up to 2.5 times higher climate impacts than plastic. Cost-wise, bamboo straws reached EUR 7.97/year, while silicone and metal straws were more economically favorable at EUR 1.17 and EUR 2.81, respectively. The consumer survey highlighted that 85% of users preferred traditional plastic straws, but 76% were open to reusable alternatives. From a socio-economic point of view, cost seems to play a minor role. However, consumers’ preferences towards the new products and their awareness of health and environmental risks are very significant factors affecting their approval of new alternatives and their displeasure towards traditional straw elimination.

## 1. Introduction

The wide use of over 30 billion straws annually in the European Union (EU) in 2020 highlights the extensive reliance on single-use plastics, despite growing awareness of their environmental and health impacts [1]. These straws, often used for mere minutes, contribute significantly to plastic pollution, which affects ecosystems, wildlife, and human health on a global scale. Single-use plastics like straws can take hundreds of years to decompose, often breaking down into microplastics that infiltrate water sources, soil, and even the food chain [2]. Moreover, the production and disposal of these plastics release greenhouse gases, exacerbating climate change [3]. Recognizing these dangers, the EU implemented a ban on single-use plastics in 2021, aiming to reduce plastic waste and promote sustainable alternatives [4] that are less harmful to the environment. This shift underscores the urgent need for collective action to address plastic pollution, emphasizing the importance of reducing consumption, improving waste management, and adopting eco-friendly practices to safeguard the planet and future generations.

Reusable straws made from materials like stainless steel, bamboo, and glass have gained popularity as durable and eco-friendly options [4]. Additionally, biodegradable straws made from paper, hay, or plant-based materials such as PLA (polylactic acid) offer a compostable solution that breaks down naturally without leaving microplastics [2]. However, PLA straws, though biobased, are banned under the SUP Directive unless proven to meet specific compostability standards (EN 13432 [5]). Businesses and consumers are increasingly embracing these alternatives, driven by both regulatory requirements and growing environmental awareness [6]. Innovations in material science have also led to the development of edible straws, made from rice, seaweed, or other food-grade ingredients, which eliminate waste entirely [3]. Governments and organizations are supporting this transition through public awareness campaigns, subsidies for sustainable products, and partnerships with eco-conscious manufacturers [4]. By promoting these alternatives, the EU is fostering a circular economy that prioritizes resource efficiency and minimizes environmental impact. This shift not only addresses plastic pollution but also sets a global example for sustainable consumption and production practices, paving the way for a greener future.

While sustainable alternatives to single-use plastics, such as reusable, biodegradable, or even edible straws, are often promoted as environmentally superior, their advantages over conventional plastic straws can be vague and context dependent. It is worth noting that biodegradability alone does not exempt materials from the SUP Directive; natural polymers (e.g., PHA) may qualify if non-synthetic. In addition, reusable straws made from materials like stainless steel or silicone require significant energy and resources to produce, and their environmental benefits only materialize after repeated use, which may not always be guaranteed due to consumer behavior [7]. Biodegradable straws, such as those made from PLA or paper, are often touted as eco-friendly, but their benefits hinge on proper disposal and access to industrial composting facilities, which are not universally available [8]. Without such infrastructure, these straws may end up in landfills or natural environments, where they degrade slowly or release microplastics, undermining their perceived advantages. Additionally, the production of plant-based alternatives can still have environmental costs, such as land and water use for growing raw materials like bamboo or corn [3]. Furthermore, the carbon footprint of transporting these alternatives, often manufactured in specific regions, can offset some of their environmental gains. Thus, while sustainable alternatives hold promise, their overall advantage over conventional plastics is not always clear-cut and depends heavily on factors like production methods, consumer habits, and waste management systems [9,10]. This vagueness underscores the need for comprehensive lifecycle assessments and systemic changes to fully realize the potential of these alternatives.

**Figure 1:**
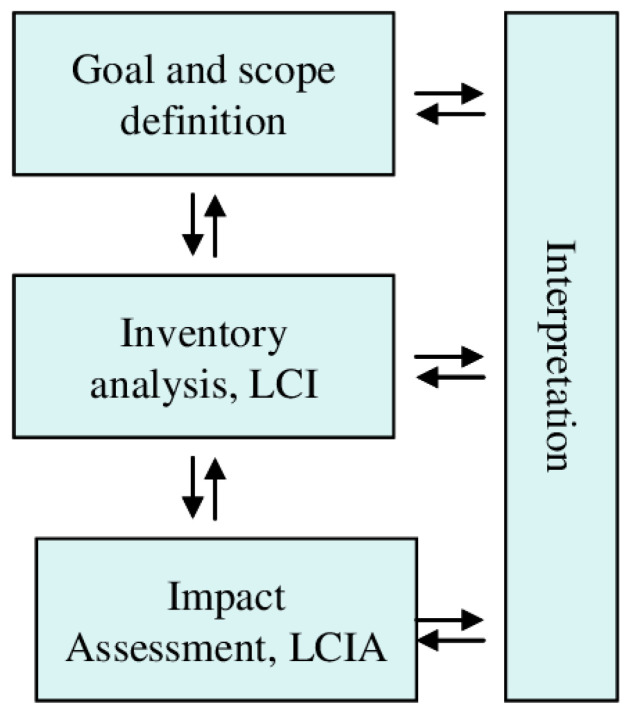
LCA framework (ISO 14040, 2006) [11].

Life-cycle assessment (LCA) is a systematic methodology used to evaluate the environmental impacts of a product, process, or system throughout its entire lifecycle, from raw material extraction to disposal or recycling. It provides a comprehensive framework for comparing the environmental performance of different systems, such as conventional single-use plastics and their sustainable alternatives. LCA typically involves four main stages: (1) goal and scope definition, where the purpose and boundaries of the study are established; (2) life cycle inventory (LCI), which quantifies the energy, materials, and emissions associated with each stage of the lifecycle; (3) life cycle impact assessment (LCIA), where the inventory data are translated into environmental impacts, such as global warming potential, resource depletion, or water use; and (4) interpretation, where the results are analyzed to draw conclusions and inform decision making (Figure 1) [11].

When applied to single-use plastics and their alternatives, LCA can reveal insights that go beyond simplistic comparisons. For example, while reusable straws made from stainless steel or silicone may have higher initial environmental impacts due to energy-intensive production, their repeated use over time can result in lower overall impacts compared to single-use plastic straws [7]. Similarly, biodegradable straws made from PLA or paper may show lower carbon footprints in the production phase, but their environmental benefits depend on end-of-life scenarios, such as whether they are composted, mechanically recycled, or end up in landfills [8]. LCA can also highlight trade-offs between different impact categories; for instance, plant-based alternatives might reduce plastic pollution but increase water or land use due to agricultural demands [3]. By quantifying these trade-offs, LCA enables policymakers, businesses, and consumers to make informed choices that balance environmental priorities and practical considerations.

The purpose of this article is the implementation of LCA to these alternative systems, such as sustainable straws, in order to provide a scientifically rigorous and holistic understanding of their environmental effectiveness compared to conventional single-use plastics. By systematically analyzing the entire lifecycle of these alternatives—from raw material extraction and production to use, disposal, and potential recycling—the article aims to identify key environmental impacts, trade-offs, and opportunities for improvement. This approach helps to move beyond “simple” claims about sustainability, offering data-driven conclusions that can inform policymakers, businesses, and consumers. The article will highlight areas where further innovation or systemic changes are needed, such as improving the efficiency of production processes or expanding waste management capabilities. Ultimately, the goal is to provide a transparent, evidence-based foundation for decision making, enabling stakeholders to adopt solutions that genuinely reduce environmental harm and contribute to a more sustainable future. By doing so, the article not only advances academic understanding but also supports practical efforts to address the global challenge of plastic pollution.

Finally, apart from the LCA, a preliminary cost analysis and a consumer preferences survey were conducted to assess the feasibility and acceptance of these alternatives to single-use plastics. The cost analysis examined the total cost for the end-user for a whole year of use. The consumer preferences survey aimed to gauge public opinion on the use of alternative materials, such as paper, metal, or bamboo straws, and their willingness to adopt these alternatives despite potential higher costs, practicality issues, and convenience of switching to new materials. These insights are also crucial for understanding how to, effectively, implement changes in consumer behavior and industry practices, ensuring that the shift to sustainable alternatives is both economically viable and widely accepted.

## 2. Methodology

### 2.1. Goal and Scope

Goal: In the context of comparing conventional single-use plastic straws with sustainable alternatives, the goal of the LCA study would be to quantify and compare the environmental burdens of each option across various impact categories, such as global warming potential, resource depletion, water use, and waste generation. This comparison aims to identify which system performs better environmentally and under what conditions, while also highlighting potential trade-offs and areas for improvement. By adhering to the ISO 14040 framework [11], the study ensures that the analysis is transparent, reproducible, and based on scientifically robust data, thereby providing stakeholders with reliable information to make informed decisions. The ultimate objective is to support the development and adoption of more sustainable practices and products, contributing to the reduction in environmental impacts and the promotion of a circular economy.

Scope: According to ISO 14040 [11], the scope of a life-cycle assessment (LCA) must clearly define several key items to ensure the study is transparent, consistent, and fit for its intended purpose. These items are described in the below paragraphs.

### 2.2. Function Unit (FU)

The functional unit for this comparative study is defined as the number of straws used by a single person over a year. In Greece, the average annual consumption per person is estimated to be approximately 50 single-use plastic straws, based on responses from the consumer preference survey. This number may vary across different countries and continents where single-use plastic bans are not in place. For instance, in the USA and Malaysia, the estimated daily consumption is 1.6 and 1 straw per capita, respectively [12]. Consequently, for single-use straws, the functional unit is set to a few dozen, while for reusable straws, this is set to just a few units (or a single unit). Details on the FUs used are presented in Table 1.

### 2.3. Systems Boundaries

**Figure 2:**
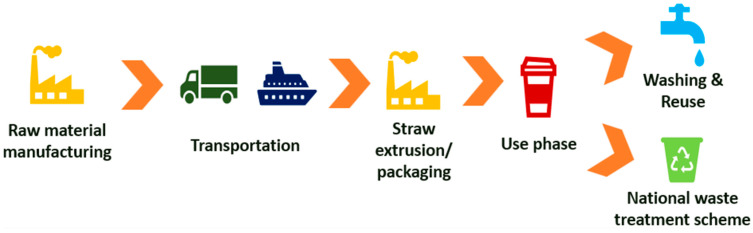
Overall system boundary of straws from gate to grave.

For the studied systems, a cradle-to-grave approach is adopted. This assessment encompasses all stages of the product lifecycle, from raw material extraction (“cradle”) to final disposal or recycling (“grave”). Key stages included within these boundaries are: (1) raw material extraction, which covers the sourcing of materials like petroleum for plastic straws or bamboo for reusable alternatives; (2) production, including processes like extrusion, molding, or manufacturing; (3) packaging, which accounts for the materials and energy used to package the straws for distribution; (4) transportation, covering the movement of materials and finished products from factories to retailers or consumers; (5) retail and use phase, which considers the energy and resources associated with storage, display, and consumer use; and (6) end-of-life (EoL), including disposal methods such as landfilling, incineration, composting, or recycling, as well as potential reuse for durable alternatives (Figure 2). By including all these stages, the LCA provides a comprehensive evaluation of the environmental impacts associated with each system, ensuring that no significant contributions are overlooked. This holistic approach allows for a fair and accurate comparison between conventional and sustainable alternatives, highlighting trade-offs and opportunities for reducing environmental burdens across the entire lifecycle.

### 2.4. Product Systems

In this study, the environmental impacts of nine distinct product systems (straws) within the context of Greece in 2024 are examined. The base system for comparison is the conventional single-use plastic straw made from polypropylene (PP), which remains widely used despite growing environmental concerns.

Apart from this, eight alternative systems are evaluated, encompassing a range of materials and designs aimed at reducing environmental harm. These alternatives include reusable straws made from:

- stainless steel
- silicone
- glass
- bamboo

as well as biodegradable options such as

- paper
- polylactic acid, PLA
- polyhydroxyalkanoates, PHA
- wheat

Each system is assessed across its entire lifecycle—from raw material extraction and production to packaging, transportation, use, and end-of-life disposal or reuse—to provide a comprehensive comparison of their environmental performance. By focusing on the Greek region, the study considers local factors such as waste management infrastructure, consumer behavior, and transportation networks, ensuring that the findings are relevant and actionable for policymakers, businesses, and consumers in Greece. This analysis aims to identify the most sustainable options for reducing plastic waste and promoting eco-friendly practices in the region. Details for each product system are presented in Table 1.

Apart from the product systems, a significant process that must be acknowledged is the reuse phase of the reusable alternatives. For the energy, water, and material consumption for this phase, three different scenarios have been considered, and they are summarized in Table 2 [10,13,14].

### 2.5. Data Requirements [15,16]

The data requirements for this LCA study focus on quantifying the material inputs, energy use and water consumption, and waste treatment for each examined straw system. Specifically, necessary data for raw materials, production processes (e.g., extrusion, molding), packaging, transportation (modes, distances), use phase (including reuse for durable alternatives), and EoL scenarios such as waste treatment (e.g., landfilling, incineration, composting, or recycling) are collected from both **primary data** (data derived directly from common commercial products such as weight, dimensions, type of materials, origin, etc.) and **secondary data** (from peer-reviewed literature and/or established databases such as European Life Cycle Database (ELCD) and Ecoinvent) to ensure accuracy and consistency. These sources provide reliable, standardized information on resource use, emissions, and energy consumption, enabling a robust comparison of the environmental performance of conventional and alternative straw systems.

### 2.6. Assumptions and Limitation

Key assumptions, that must be acknowledged, include:

1. average consumer behavior regarding:
  (a) the number of straws used per year (set to 50 for single-use straws)
  (b) times reusable straws are used and washed before disposal (set to infinite for stainless steel and silicone straws, to 25 for glass straws and to 10 for bamboo),
  (c) washing and reuse scenarios (see Table 2)
2. standard transportation distances and modes, based on typical supply chains in Greece, and
3. uniform waste treatment scenarios (e.g., landfilling, composting, or recycling) for Greece (details for each straw treatment are presented in Table 1).

Additionally, it is assumed that all materials and processes are representative of industry averages, as specific data from local manufacturers were not available. Thus, limitations of the study include the reliance on secondary data from literature and databases, which may not fully capture regional variations.

### 2.7. Impact Categories and Method

The study utilizes the ReCiPe 2016 H (Hierarchist) impact assessment method to evaluate the environmental impacts of the examined straw systems. ReCiPe 2016 is a widely recognized and comprehensive life cycle impact assessment (LCIA) method that integrates midpoint (18) and endpoint (3) approaches, providing a holistic view of environmental impacts. The hierarchist perspective is chosen as it represents a balanced view, combining scientific consensus and societal preferences, making it suitable for policy and decision-making contexts. For better representation of the results, this study focuses on 12 impact categories (9 midpoints and 3 endpoints); however, all results are available in the Supplementary Materials. These categories are:

- Climate change (exld. Biogenic CO_2_) [CC]
- Fine particulate matter formation [FPMF]
- Fossil depletion [FD]
- Freshwater consumption [FWC]
- Freshwater ecotoxicity [FEW]
- Freshwater eutrophication [FEW]
- Human toxicity, cancer [HT]
- Metal depletion [MD]
- Terrestrial acidification

⮚ Damage to human health [DHH]
⮚ Damage to ecosystems [DES]
⮚ Damage to resource availability [DRA]

By applying ReCiPe 2016 H, the study quantifies the environmental burdens associated with each straw type across its lifecycle, from raw material extraction to disposal, enabling a robust comparison of their sustainability. This approach ensures that the results are scientifically rigorous and aligned with contemporary environmental priorities, providing actionable insights for stakeholders aiming to reduce the environmental footprint of single-use and reusable straws.

### 2.8. Life Cycle Inventory (LCI)

The LCI for this study provides a comprehensive breakdown of the materials, energy, and processes involved in the production, use, and disposal of each straw type. Detailed data for each product, including raw material inputs, manufacturing processes, transportation, and end-of-life scenarios, are documented and presented in the Supplementary Materials (Tables S1–S9). This includes specific quantities of materials (such as polypropylene for plastic straws, kraft paper for paper straws, PLA and PHA for bioplastic straws, and stainless steel and glass for reusable straws, etc.) as well as energy and water consumption during production and washing. The Supplementary Materials also outline assumptions, such as the functional unit equivalence of 50 single-use straws per person and year for comparison with one reusable straw. By providing these data, the study ensures transparency and reproducibility, allowing stakeholders to verify the results and apply the findings to their own sustainability assessments or policy decisions.

### 2.9. Consumer Preference Survey

**Figure 3:**
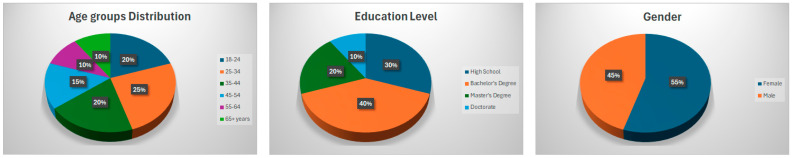
Demographic data of participants in consumer preference survey.

A consumer preference survey was conducted to assess the impact of the ban on single-use plastics, specifically focusing on plastic straws. The survey was administered to a sample of 100 participants, selected to represent a diverse cross-section of the Greek population in terms of age (18–65+), gender (55% female, 45% male), geographic distribution (urban/rural), and socio-economic status (income, education). Participants were recruited via email to ensure balanced demographic coverage, as visualized in Figure 3. The questionnaire, pilot-tested for clarity, included questions about straw usage habits, material preferences, and environmental perceptions. Online administration facilitated rapid data collection while maintaining accessibility across different demographic groups.

Survey questions are provided as Supplementary Materials (Table S11).

## 3. Results and Discussion

### 3.1. Life Cycle Impact Assessment (LCIA)

**Figure 4:**
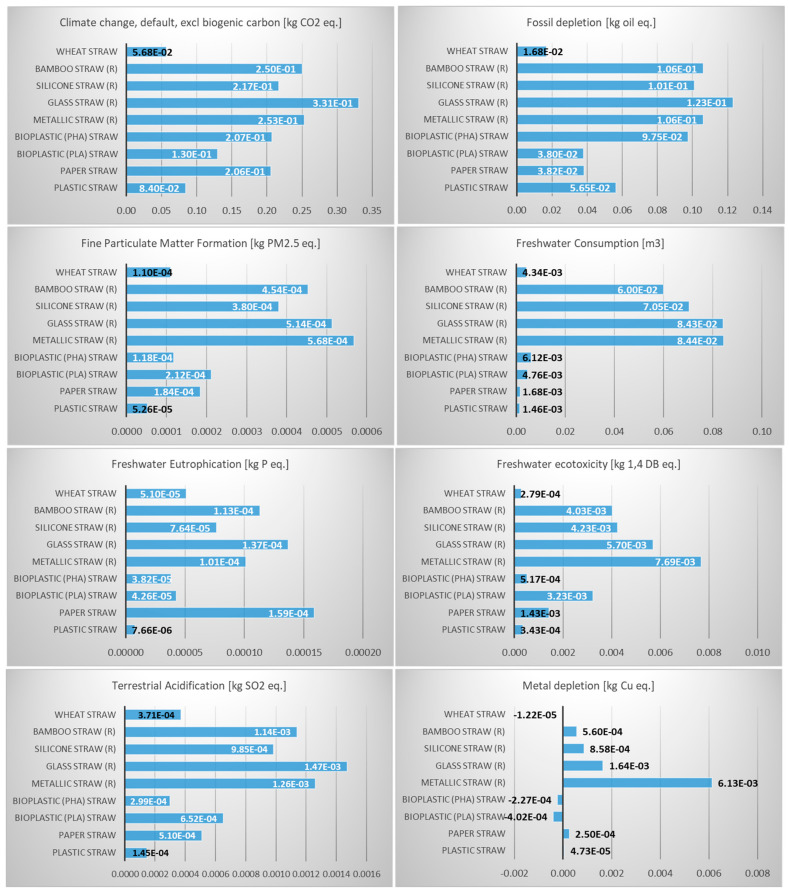

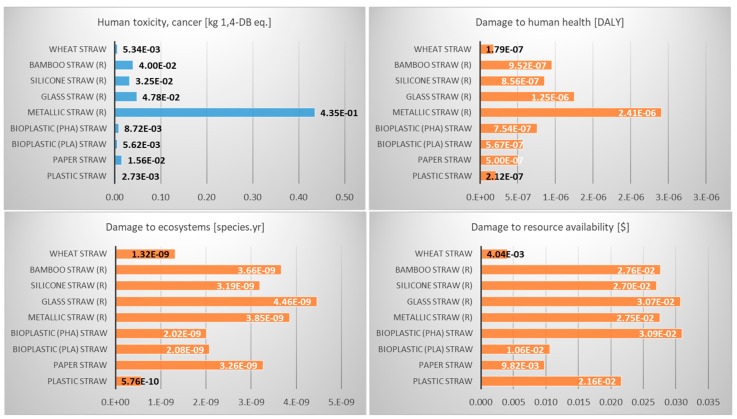
LCIA results on the environmental performance of the 9 examined product systems (R: reusable, impact categories in blue for the 9 midpoints and in orange for the 3 endpoints).

The life cycle impact assessment (LCIA) results provide a comprehensive comparison of the environmental impacts of various straw types across multiple impact categories. The LCIA results for the nine examined product systems (50 uses per year, hand wash with cold water) are presented in Figure 4. Detailed results for all impact categories are presented in Table S10 in the Supplementary Materials.

While this study focuses on the polymer matrix (PP), it is acknowledged that plastic straws often contain additives (e.g., plasticizers, colorants) that may leach into the environment during degradation. These additives can exacerbate toxicity impacts (e.g., freshwater ecotoxicity in Table S10), though quantitative data were excluded due to lack of standardized LCI datasets for Greek market-specific formulations. Future studies should incorporate additive-related impacts when region-specific data become available.

Notably, plastic straws exhibit very low impacts in several categories, including climate change (both excluding and including biogenic carbon), freshwater consumption, and damage to human health. However, their low impact in these categories is offset by significant contributions to fossil depletion and terrestrial ecotoxicity, highlighting the reliance on non-renewable resources and potential long-term environmental harm. In contrast, wheat straws emerge as a strong alternative, demonstrating the lowest impact on climate change (including biogenic carbon), freshwater ecotoxicity, and damage to resource availability. This suggests that wheat straws, as a natural and renewable material, may offer a more sustainable option, particularly in terms of resource efficiency and ecosystem preservation.

Reusable straws, such as metallic, glass, silicone, and bamboo straws, generally show higher impacts in categories like freshwater consumption, human toxicity, and terrestrial ecotoxicity. This is likely due to the energy-intensive manufacturing processes and resource extraction required for their production. For instance, metallic straws have the highest impact on human toxicity (cancer) and freshwater consumption, while glass straws score poorly in fossil depletion and freshwater ecotoxicity. Despite their reusability, these straws may only become environmentally favorable after many uses, as their initial production impacts are significantly higher than single-use alternatives. On the other hand, bioplastic straws (PLA and PHA) show mixed results. While they perform better than paper straws in some categories, such as climate change (excluding biogenic carbon), they have higher impacts in freshwater consumption and human toxicity (non-cancer), indicating that their sustainability claims may be context dependent.

**Figure 5:**
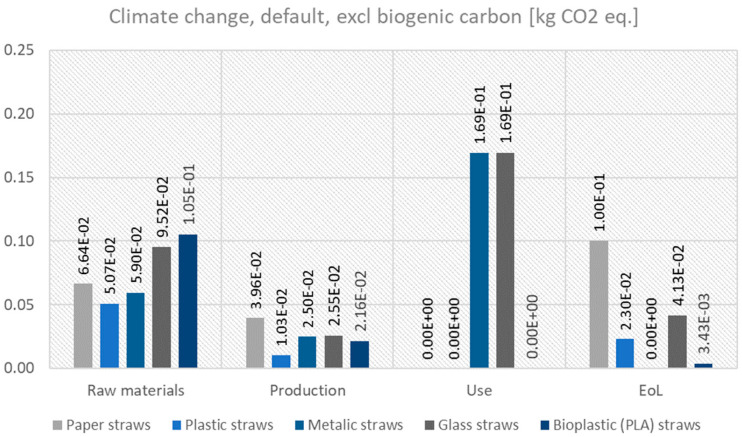
LCIA results on the climate change impact category, along the different stages of the life cycle of some of the examined straws.

In Figure 5, the contribution of each life cycle stage to the total climate change impact category of several examined straws is presented.

The breakdown of climate change impacts (excluding biogenic carbon) across the lifecycle stages of paper, plastic, metallic, bioplastic, and glass straws reveals significant differences in where the environmental burdens occur.

For **paper straws**, the majority of the climate change impact arises during the **raw materials** (6.64 × 10^−2^ kg CO_2_ eq.) and **end-of-life (EoL)** stages (1.00 × 10^−1^ kg CO_2_ eq.), with a smaller contribution from production (3.96 × 10^−2^ kg CO_2_ eq.). This suggests that the sourcing of raw materials (e.g., forestry and pulp processing) and disposal (e.g., decomposition or incineration) are the primary drivers of emissions. The absence of emissions during the use phase reflects the single-use nature of paper straws.

In contrast, **plastic straws** show a lower overall climate change impact, with the largest contribution coming from the **raw materials** stage (5.07 × 10^−2^ kg CO_2_ eq.), followed by production (1.03 × 10^−2^ kg CO_2_ eq.) and EoL (2.30 × 10^−2^ kg CO_2_ eq.). The relatively low impact of plastic straws in this category is consistent with their lightweight and energy-efficient production, though their persistence in the environment and reliance on fossil fuels remain significant concerns. The absence of emissions during use is again due to their single-use design.

For **metallic straws**, the **use phase** dominates the climate change impact (1.69 × 10^−1^ kg CO_2_ eq.), likely due to the energy required for cleaning over their lifecycle. The raw materials (5.90 × 10^−2^ kg CO_2_ eq.) and production (2.50 × 10^−2^ kg CO_2_ eq.) stages also contribute significantly, reflecting the energy-intensive extraction and processing of metals. The absence of EoL impacts suggests that metallic straws are assumed to have a negligible end-of-life footprint, possibly due to very long-term reuse.

Similarly, **glass straws** exhibit a high climate change impact during the **use phase** (1.69 × 10^−1^ kg CO_2_ eq.), again tied to the cleaning process. The raw materials (9.52 × 10^−2^ kg CO_2_ eq.) and production (2.55 × 10^−2^ kg CO_2_ eq.) stages also contribute, with a notable EoL impact (4.13 × 10^−2^ kg CO_2_ eq.) due to the disposal. This highlights the trade-off between the durability of reusable straws and the energy demands associated with their lifecycle.

These results underscore the importance of considering the full lifecycle when evaluating straw alternatives. While **paper straws** have high raw material and EoL impacts, **plastic straws** show lower emissions but raise concerns about resource depletion and pollution. **Metallic and glass straws**, despite their reusability, incur significant climate change impacts during use and production, emphasizing the need for energy-efficient cleaning methods and sustainable material sourcing to maximize their environmental benefits.

### 3.2. Interpretation

The number of uses per person per year is a critical factor in interpreting life-cycle assessment (LCA) results, as it directly influences the overall environmental impact of reusable versus single-use straws. For single-use straws like paper, plastic, and bioplastic (PLA), the impact is linear with usage—each straw is used once and then discarded, meaning that 50 uses per year result in 50 individual straws being produced, used, and disposed of. This linear relationship makes their environmental footprint highly sensitive to the number of uses, as higher usage rates lead to proportionally higher impacts in raw material extraction, production, and end-of-life stages. For reusable straws such as metallic, glass, silicone, and bamboo, the environmental impact is heavily front-loaded in the raw materials and production stages due to their more resource-intensive manufacturing processes. However, their impact per use decreases significantly with repeated use.

**Figure 6:**
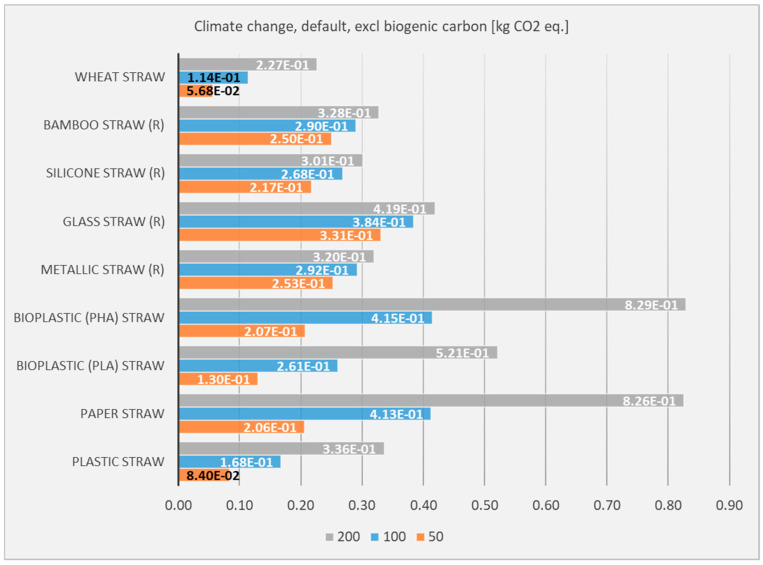
LCIA results on the climate change impact category, regarding the number of uses per person and year (50, 100, and 200 uses).

In order to examine this effect, different scenarios of uses per person have been examined. Apart from the one that has already been examined, two more (100 and 200) have been calculated and presented in Figure 6.

The number of uses per person per year seems to be a key determinant in the sustainability of straws. For reusable straws to be environmentally preferable, they must be used frequently enough to justify their higher initial impacts. However, single-use straws, while having lower per-unit impacts, accumulate significant environmental burdens with increased usage. Policymakers and consumers should consider usage patterns when choosing straw types, as the optimal choice depends on how often the straw will be used and the efficiency of its lifecycle management.

Another significant factor (as suggested from Figure 4) that determines the environmental impacts of the reusable straws is the use (cleaning) phase. The scenario analysis of Table 2 for washing reusable straws highlights the environmental trade-offs associated with different cleaning methods: hand washing with cold water, hand washing with hot water, and using a dishwasher. Each method has distinct implications for water, energy, and detergent consumption, which directly influence the overall environmental footprint of reusable straws.

**Figure 7:**
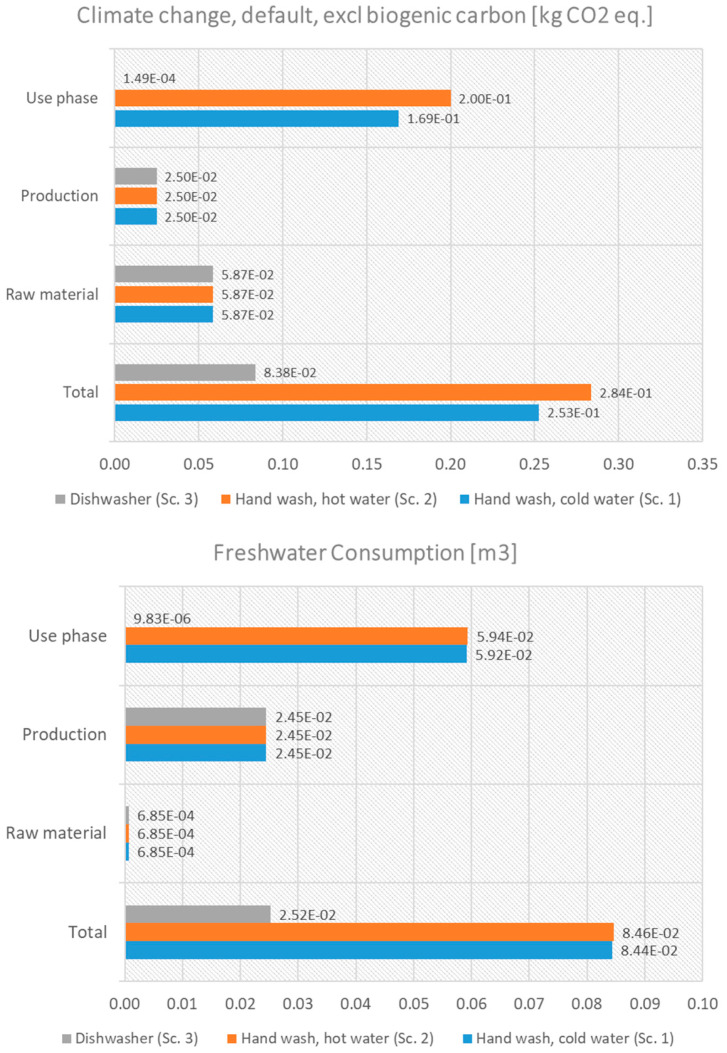
Effect of different washing methods on climate change and freshwater consumption impact categories for metallic straws.

Figure 7 presents the effect of these different washing methods on climate change and freshwater consumption impact categories for metallic straws. The same picture is also shown for other categories, such as freshwater ecotoxicity and eutrophication. As Figure 6 suggests, dishwashing is likely the most sustainable option due to its water and detergent efficiency, while energy consumption is relatively small. Hand washing with cold water is a viable option for regions with abundant water resources, but detergent use should be minimized. Hand washing with hot water is the worst scenario among the studied ones.

Overall, the results underscore the complexity of evaluating environmental impacts, as no single straw type performs optimally across all categories. Wheat straws stand out as a promising low-impact option, particularly for single-use scenarios, while reusable straws may require careful consideration of their lifecycle impacts and usage frequency to justify their environmental benefits. The data also highlights the importance of considering biogenic carbon and resource availability in LCIA, as these factors significantly influence the comparative sustainability of different materials.

### 3.3. Preliminary Cost Analysis

Preliminary cost calculations for the different types of straws—ranging from single-use options like plastic, paper, and bioplastic (PLA) to reusable alternatives such as metallic, glass, silicone, and bamboo—are essential for understanding their economic viability alongside their environmental impacts. While life-cycle assessment (LCA) provides insights into the ecological footprint of each straw type, a cost analysis complements this by evaluating the financial implications of their use.

For the preliminary calculations, the basis of 50 uses of straws per person and year has been taken into account. For the use phase, the dishwash scenario has been selected. No costs for the EoL phase have been assigned. The unit costs have been obtained from the Greek market (electricity cost: 0.25 EUR/kWh, water cost: 0.001 EUR/L, dishwasher detergent cost: 5 EUR/L).

The cost analysis reflects 2024 Greek market prices, which are sensitive to global supply chains (e.g., wheat straws imported from China). However, economies of scale could reduce costs for local production of alternatives (e.g., PHA from fruit waste), as seen in the EU’s bioeconomy strategy [17]. This aligns with findings in Table 3, where reusable straws’ long-term cost efficiency is less volatile due to durability.

The obtained results are presented in Table 3.

As far as single-use straws are concerned, plastic straws are the cheapest solution. Wheat straws, while eco-friendly, are very expensive since they are currently imported from China. Paper and bioplastic straws come at a higher cost while their sustainability benefits are not clear.

For reusable straws, the metallic and silicone ones are the most cost-effective reusable options over time, with low reuse costs and high durability. Glass straws may be considered less economical due to their fragility, while bamboo straws are the least cost-effective due to their short lifespan while they are currently imported from China.

### 3.4. Consumer Preference Survey

Consumers habits have been significantly altered after the ban on single-use plastics. One of the basic questions of the survey was to indicate the number of times they are using straws in their daily lives. This number was, on average, 50 straws per year, which is the number considered as the basis for the FU used in PP straws. Among this important information, consumer acceptance questionnaires revealed very interesting results: (a) 85% feel more comfortable using traditional PP straws, although 75% believe they have the highest climate impact; (b) 92% consider paper straws the most inappropriate, despite all participants recognizing them as the most cost-effective option; (c) 76% think reusable straws could be a good alternative to PP straws, but 95% are not willing to use silicone straws, while 68% prefer metallic straws and the remainder prefer glass straws.

Another important outcome from this survey is that 95% of participants believe the ban on PP straws is due to their non-recyclability, rather than the fact that their mass and collection routes currently hinder recycling with available techniques and practices.

## 4. Conclusions

The comparative analysis of straw types—ranging from **single-use options like plastic, paper, and bioplastics** to **reusable alternatives** such as **metallic, glass, silicone, and bamboo**—reveals a complex interplay between economic costs and environmental impacts. Single-use straws, particularly **plastic**, are the most economical in terms of upfront costs but come with significant environmental drawbacks, including pollution, resource depletion, and long-term ecological harm. **Paper** and **bioplastic** straws, while more sustainable in some respects, are more expensive and may still contribute to environmental degradation through resource-intensive production processes. **Wheat** straws, though natural and biodegradable, are the least economically viable single-use option due to their high cost. On the other hand, **reusable straws**, such as **metallic** and **silicone**, present a compelling case for long-term sustainability. Despite their higher initial costs, their durability and low reuse expenses make them cost-effective over time while significantly reducing waste and resource consumption.

Ultimately, the choice of straw type depends on balancing economic feasibility with environmental priorities. For consumers and businesses aiming to minimize costs, metallic or silicone straws offer the best combination of affordability and sustainability. For those prioritizing immediate environmental benefits, bioplastic or wheat straws may be preferable, though their higher costs and limited availability must be considered.

In conclusion, within the limitations of this paper, traditional polypropylene (PP) straws were found to be the most feasible and appealing option for consumers. This aligns with comparisons of other petroleum-based products and their alternatives due to optimized procedures throughout their entire lifecycle. A lesser-known fact is that these plastic straws cannot be recycled due to their small weight. Consequently, the end-of-life disposal of these non-recyclable products contributes to their poor environmental performance, with well-documented consequences and their share of blame for plastic pollution in seas, land, and other ecosystems. As shown, other single-use straw options are similarly, if not more, detrimental from an environmental perspective. Therefore, it is worth considering whether the scenario of mass collection and pretreatment of PP straws could make their recycling feasible, rather than imposing a ban without first examining the proposed alternatives [18,19].

## Figures and Tables

**Table 1:** Process parameters of the examined product systems.

Product System									
	Base	1	2	3	4	5	6	7	8
**Basic data**									
Description	Plastic straw	Paper straw	Biopolymer straw	Biopolymer straw	Wheat straw	Metallic straw	Glass straw	Silicone straw	Bamboo straw
Picture	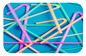	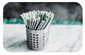	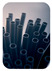	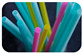	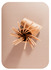	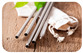	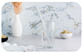	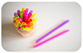	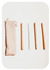
Material	PP	Paper	PLA (from corn starch)	PHA (from fruit waste)	Wheat	SS304	Borosilicate Glass	Silicone	Bamboo stem
Dimensions LxD (mm)	210 x ∅ 5.5	210 x ∅ 6	210 x ∅ 6	210 x ∅ 6	200 x ∅ 4	200 x ∅ 6	220 x ∅ 9	250 x ∅ 9	200 x ∅ 12
Weight (g/pcs)	0.5	2.5	0.9	0.9	0.7	11.0	23.0	12.0	9.0
**[FU] No of pieces** **(per year, per person)**	**50**	**50**	**50**	**50**	**50**	**1**	**2**	**1**	**5**
Origin	Italy	Germany	Belgium	Italy	China	Greece	Greece	China	China
End-of-life (EoL)	Landfill Incineration Recycle	Landfill Incineration Recycle	Compost	Compost	Compost	No	Landfill Incineration Recycle	No	Compost
**Packaging**									
Type	Plastic film	Paper film	Paper film	Paper film	Cardboard box	Textile bag	Textile bag	Textile bag	Cardboard box
Material	LDPE	Paper	Paper	Paper	Paper	Cotton clothing	Cotton clothing	Cotton clothing	Paper
Weight (g/pcs)	0.1	0.12	0.12	0.12	40	11	11	11	35
Straws per package	1	1	1	1	100	2	4	5	6
Other packaged items	*No*	*No*	*No*	*No*	*No*	*Brush*	*Brush*	*Brush*	*No*
Material 1						Wire, 5 g	Wire, 5 g	Wire, 5 g	
Material 2						Nylon, 0.3 g	Nylon, 0.3 g	Nylon, 0.3 g	
Straws in packaging						2	4	5	
**Use Phase**									
Purpose	*Single*	Single	Single	Single	Single	Reusable	Reusable	Reusable	Reusable
Uses (per year)	*1*	1	1	1	1	50	50	50	50
Additional need	*No*	No	No	No	No	Washing	Washing	Washing	Washing

**Table 2:** Reuse scenarios and requirements (CCW, n.d.) (Rana, 2020) [13].

Scenario	Unit	1	2	Unit	3
Description		Washed by hand Cold water (17 °C), 0.1% detergent	Washed by hand Hot water (50 °C), 0.1% detergent		Dishwasher (Cycle: 6 kg dishes, 1 kwh electricity, 20 L water, 80 g detergent)
Water	[L/straw/wash]	1	0.9	[L/g straw/cycle]	0.00333
Energy	[kWh/straw/wash]	0.000	0.06	[kWh/g straw/cycle]	0.00017
Detergent	[g/straw/wash]	1	1	[g/g straw/cycle]	0.01333

**Table 3:** Preliminary cost analysis (per person and year).

	Unit Cost [€/pcs]	Weight (gr/pcs)	No of Straws per Year	Use	No of Uses	Purchase Cost [€]	Reuse Cost [€]	Total Cost [€]
**Plastic straw**	0.006		50	S	1	0.30		**0.30**
**Paper straw**	0.014		50	S	1	0.70		**0.70**
**Bioplastic (PLA) straw**	0.026		50	S	1	1.30		**1.30**
**Bioplastic (PHA) straw**	0.015		50	S	1	0.75		**0.75**
**Metallic straw**	2.750	11.00	1	R	50	2.75	0.064	**2.81**
**Glass Straw**	1.000	23.00	2	R	25	2.00	0.133	**2.13**
**Silicone straw**	1.100	12.00	1	R	50	1.10	0.070	**1.17**
**Bamboo straw**	1.583	9.00	5	R	10	7.92	0.052	**7.97**
**Wheat straw**	0.070		50	S	1	3.50		**3.50**

## Data Availability

The original contributions presented in this study are included in the article/Supplementary Materials. Further inquiries can be directed to the corresponding author(s).

## Supplementary Materials

The following supporting information can be downloaded at: https://www.mdpi.com/article/10.3390/polym17091235/s1, Table S1. Inventory data for conventional plastic straws (per person and year); Table S2. Inventory data for paper straws (per person and year); Table S3. Inventory data for bioplastic straws (PLA) (per person and year); Table S4. Inventory data for bioplastic straws (PHA) (per person and year); Table S5. Inventory data for metallic straws (Stainless steel); Table S6. Inventory data for glass straws; Table S7. Inventory data for silicone straws; Table S8. Inventory data for biobased straws (bamboo) (per person and year); Table S9. Inventory data for biobased straws (wheat) (per person and year); Table S10. LCIA results for the examined systems (50 uses per person and year); Table S11. Consumers’ preference survey Questions.

## References

[1] European Commission (2018). Single-Use Plastics: New EU Rules to Reduce Marine Litter. https://ec.europa.eu/commission/presscorner/detail/en/ip_18_3927.

[2] UNEP—United Nations Environment Programme (2018). Single-Use Plastics: A Roadmap for Sustainability.

[3] Geyer R., Jambeck J.R., Law K.L. (2017). Production, use, and fate of all plastics ever made. Sci. Adv..

[4] The European Parliament and the Council of the European Union (2019). Directive (EU) 2019/904 of the European Parliament and of the Council of 5 June 2019 on the Reduction of the Impact of Certain Plastic Products on the Environment. https://eur-lex.europa.eu/eli/dir/2019/904/oj/eng.

[5] (2000). Packaging. Requirements for Packaging Recoverable Through Composting and Biodegradation. Test Scheme and Evaluation Criteria for the Final Acceptance of Packaging.

[6] Parker L. (2019). The World’s Plastic Pollution Crisis, Explained. https://www.nationalgeographic.com/environment/article/plastic-pollution.

[7] George Bishop D.S. (2021). Environmental performance comparison of bioplastics and petrochemical plastics: A review of life cycle assessment (LCA) methodological decisions. Resour. Conserv. Recycl..

[8] Adele Folino A.K. (2020). Biodegradation of Wasted Bioplastics in Natural and Industrial Environments: A Review. Sustainability.

[9] European Environment Agency (2020). Biodegradable and Compostable Plastics—Challenges and Opportunities. https://www.eea.europa.eu/en/analysis/publications/biodegradable-and-compostable-plastics.

[10] How Much Water Do You Use?. https://www.ccw.org.uk/save-money-and-water/averagewateruse/.

[11] (2006). Environmental Management—Life Cycle Assessment—Principles and Framework.

[12] Moy C.-H., Tan L.-S., Shoparwe N., Shariff A., Tan J. (2021). Comparative Study of a Life Cycle Assessment for Bio-Plastic Straws and Paper Straws: Malaysia’s Perspective. Processes.

[13] Rana K. (2020). Plasticless: A Comparative Life-Cycle, Socio-Economic, Plasticless: A Comparative Life-Cycle, Socio-Economic, and Policy Analysis of Alternatives to Plastic Straws and Policy Analysis of Alternatives to Plastic Straw. Master’s Thesis.

[14] Poritosh R., Ashton L., Wang T., Corradini M.G., Fraser E.D.G., Thimmanagari M., Tiessan M., Bali A., Saharan K.M., Mohanty A.K. (2021). Evolution of drinking straws and their environmental, economic and societal implications. J. Clean. Prod..

[15] Ecoinvent Database (2023). Life Cycle Inventory Data for Materials and Processes.

[16] European Commission (2020). European Life Cycle Database (ELCD).

[17] European Commission (2018). A sustainable Bioeconomy for Europe: Strengthening the Connection Between Economy, Society and the Environment. https://op.europa.eu/en/publication-detail/-/publication/edace3e3-e189-11e8-b690-01aa75ed71a1/language-en.

[18] Qiu N., Sha M., Xu X. (2022). Evaluation and future development direction of paper straw and plastic straw. IOP Conf. Ser. Earth Environ. Sci..

[19] Limpiteeprakan P., Ratchawong J., Kittichaimongkol N., Tubtimhin S. (2024). Biodegradation of bioplastic straws compared to conventional plastic straws. IOP Conf. Ser. Earth Environ. Sci..

