# Supplementary material for: Environmental and Economic Impacts of Substituting Single-Use Plastic Straws: A Life-Cycle Assessment for Greece
Source: Polymers (Basel). 2025 Apr 30;17(9):1235. doi: 10.3390/polym17091235 (PMC12074124; doi:10.3390/polym17091235)
Supplement: Supplementary file 1 [file polymers-17-01235-s001.zip › polymers-3542063-supplementary.pdf]

polymers

MDPI

Article

Environmental and Economic Impacts of Substituting Single-Use Plastic Straws: A Life-Cycle Assessment for Greece

Panagiota Eleni [1,]* and Christos Boukouvalas [2]

[1] Exelisis IKE, Consulting Company, Leof. Dekelias 215 & Skra 2, 14342 Athens, Greece
[2] School of Chemical Engineering, National Technical University of Athens, Iroon Polytechneiou 9, Zografou Campus, 15780 Athens, Greece; cbouk@chemeng.ntua.gr
* Correspondence: peny.e@exelisis.gr

Table S1. Inventory data for conventional plastic straws (per person and year).

Product system	Plastic straws (single use, PP, base)			
**Material**	**Unit**	**Value**	**Source**	**Processes**
		Production Phase		
PP	g	25	Ecoinvent	Material production, extrusion
LDPE (packaging)	g	5	Ecoinvent	Material production, film extrusion
		Transportation Phase		
Transportation 1	km	450	Ecoinvent	Ship, Italy to Greece
Transportation 2	km	1000	Ecoinvent	Truck, Italy and Greece
		Reuse Phase		
-				
		EoL Phase		
Waste	g	30	Ecoinvent	Municipal Solid Waste [GR]

Table S2. Inventory data for paper straws (per person and year).

Product system			Paper straws (single use, paper)	
**Material**	**Unit**	**Value**	**Source**	**Processes**
		Production Phase		
Paper	g	125	Ecoinvent	Production
Paper (packaging)	g	6	Ecoinvent	Production
		Transportation Phase		
Transportation 1	km	450	Ecoinvent	Ship, Italy to Greece
Transportation 2	km	2500	Ecoinvent	Truck, N. Europe to Italy and Greece
		Reuse Phase		
-				
		EoL Phase		
Waste	g	131	Ecoinvent	Municipal Solid Waste [GR]

 https://doi.org/10.3390/polym17091235

Table S3. Inventory data for bioplastic straws (PLA) (per person and year).

Product system	Bioplastic straws (single use, PLA)			
**Material**	**Unit**	**Value**	**Source**	**Processes**
Production Phase				
PLA (from corn starch)	g	45	doi.org/10.3390/polym13111854 doi.org/10.1016/j.scitotenv.2024.174349	Production
Paper (packaging)	g	6	Ecoinvent	Production
Transportation Phase				
Transportation 1 (all)	km	450	Ecoinvent	Ship, Italy to Greece
Transportation 2 (PLA)	km	1500	Ecoinvent	Truck, Belgium to Italy and Greece
Transportation 3 (paper)	km	2500	Ecoinvent	Truck, N. Europe to Italy and Greece
Reuse Phase				
-				
EoL Phase				
Waste	g	51	doi.org/10.1016/j.wasman.2023.09.004 doi.org/10.3390/su15021394	Compost [EE]

Table S4. Inventory data for bioplastic straws (PHA) (per person and year).

Product system	Bioplastic straws (single use, PHA)			
**Material**	**Unit**	**Value**	**Source**	**Processes**
Production Phase				
PHA (from fruit waste)	g	45	doi.org/10.1016/j.jclepro.2022.135331	Production
Paper (packaging)	g	6	Ecoinvent	Production
Transportation Phase				
Transportation 1 (all)	km	450	Ecoinvent	Ship, Italy to Greece
Transportation 2 (PHA)	km	850	Ecoinvent	Truck, Italy and Greece
Transportation 3 (paper)	km	2500	Ecoinvent	Truck, N. Europe to Italy and Greece
Reuse Phase				
-				
EoL Phase				
Waste	g	51	doi.org/10.1016/j.wasman.2023.09.004 doi.org/10.3390/su15021394	Compost [EE]

Table S5. Inventory data for metallic straws (Stainless steel).

**Product system**		**Metallic straws (reusable, SS304)**		
**Material**	**Unit**	**Value**	**Source**	**Processes**
Production Phase				
SS304	g	11	Ecoinvent	Pipe production
Bag (packaging)	g	5.5	Ecoinvent	Cotton fiber production
Wire (brush)	g	2.5	Ecoinvent	Steel wire
Nylon (brush)	g	0.15	Ecoinvent	Nylon 6-6
Transportation Phase				
Transportation 1 (all)	km	150	Ecoinvent	Truck, Greece
Reuse Phase				
-				*(by hand, cold water)*
Water	l	50	Ecoinvent	Tap water
Energy	kWh	0	Ecoinvent	Electricity (mix, GR)
Detergent	g	50	Ecoinvent	0.1% surfactant
EoL Phase				
Waste	g	-	-	-

Table S6. Inventory data for glass straws.

**Product system**		**Glass straws (reusable)**		
**Material**	**Unit**	**Value**	**Source**	**Processes**
Production Phase				
Glass	g	46	Ecoinvent	Borosilicate glass production
Bag (packaging)	g	5.5	Ecoinvent	Cotton fiber production
Wire (brush)	g	2.5	Ecoinvent	Steel wire
Nylon (brush)	g	0.15	Ecoinvent	Nylon 6-6
Transportation Phase				
Transportation 1 (all)	km	150	Ecoinvent	Truck, Greece
Reuse Phase				
-				*(by hand, cold water)*
Water	l	50	Ecoinvent	Tap water
Energy	kWh	0	Ecoinvent	Electricity (mix, GR)
Detergent	g	50	Ecoinvent	0.1% surfactant
EoL Phase				
Waste	g	54.15	Ecoinvent	Municipal Solid Waste [GR]

Table S7. Inventory data for silicone straws.

Product system	Silicone straws (reusable)			
**Material**	**Unit**	**Value**	**Source**	**Processes**
Production Phase				
Silicone	g	12	Ecoinvent	Silicone products
Bag (packaging)	g	2.2	Ecoinvent	Cotton fiber production
Wire (brush)	g	1	Ecoinvent	Steel wire
Nylon (brush)	g	0.06	Ecoinvent	Nylon 6-6
Transportation Phase				
Transportation 1 (all)	km	150	Ecoinvent	Truck, Greece
Transportation 2 (Silicone)	km	13000	Ecoinvent	Ship, China
Reuse Phase				
-				*(by hand, cold water)*
Water	l	50	Ecoinvent	Tap water
Energy	kWh	0	Ecoinvent	Electricity (mix, GR)
Detergent	g	50	Ecoinvent	0.1% surfactant
EoL Phase				
Waste	g	-	-	-

Table S8. Inventory data for biobased straws (bamboo) (per person and year).

Product system	Biobased straws (reusable, bamboo)			
**Material**	**Unit**	**Value**	**Source**	**Processes**
Production Phase				
Bamboo	g	45	Ecoinvent	Bamboo pole
Paper (packaging)	g	29.2	Ecoinvent	Production
Transportation Phase				
Transportation 1 (all)	km	150	Ecoinvent	Truck, Greece
Transportation 2 (all)	km	13000	Ecoinvent	Ship, China
Reuse Phase				
-				*(by hand, cold water)*
Water	l	50	Ecoinvent	Tap water
Energy	kWh	0	Ecoinvent	Electricity (mix, GR)
Detergent	g	50	Ecoinvent	0.1% surfactant
EoL Phase				
Waste	g	74.2	doi.org/10.1016/j.wasman.2023.09.004<br>doi.org/10.3390/su15021394	Compost [EE]

Table S9. Inventory data for biobased straws (wheat) (per person and year).

**Product system**	**Biobased straws (single use, wheat)**			
**Material**	**Unit**	**Value**	**Source**	**Processes**
		Production Phase		
Wheat	g	35	Ecoinvent	Wheat straws
Paper (packaging)	g	20	Ecoinvent	Production
		Transportation Phase		
Transportation 1 (all)	km	150	Ecoinvent	Truck, Greece
Transportation 2 (all)	km	13000	Ecoinvent	Ship, China
		Reuse Phase		
-				
		EoL Phase		
Waste	g	55	doi.org/10.1016/j.wasman.2023.09.004<br>doi.org/10.3390/su15021394	Compost [EE]

Table S10. LCIA results for the examined systems (50 uses per person and year).

Impact categories	Plastic straw	Paper straw	Bioplastic (PLA) straw	Bioplastic (PHA) straw	Metallic straw (R)	Glass Straw (R)	Silicone straw (R)	Bamboo straw (R)	Wheat straw
Climate change, default, excl biogenic carbon [kg CO2 eq.]	8.40E-02	2.06E-01	1.30E-01	2.07E-01	2.53E-01	3.31E-01	2.17E-01	2.50E-01	5.68E-02
Climate change, incl biogenic carbon [kg CO2 eq.]	8.89E-02	1.23E-01	2.74E-01	4.51E-01	1.61E-01	2.51E-01	1.36E-01	1.21E-01	1.64E-02
Fine Particulate Matter Formation [kg PM2.5 eq.]	5.26E-05	1.84E-04	2.12E-04	1.18E-04	5.68E-04	5.14E-04	3.80E-04	4.54E-04	1.10E-04
Fossil depletion [kg oil eq.]	5.65E-02	3.82E-02	3.80E-02	9.75E-02	1.06E-01	1.23E-01	1.01E-01	1.06E-01	1.68E-02
Freshwater Consumption [m3]	1.46E-03	1.68E-03	4.76E-03	6.12E-03	8.44E-02	8.43E-02	7.05E-02	6.00E-02	4.34E-03
Freshwater ecotoxicity [kg 1,4 DB eq.]	3.43E-04	1.43E-03	3.23E-03	5.17E-04	7.69E-03	5.70E-03	4.23E-03	4.03E-03	2.79E-04
Freshwater Eutrophication [kg P eq.]	7.66E-06	1.59E-04	4.26E-05	3.82E-05	1.01E-04	1.37E-04	7.64E-05	1.13E-04	5.10E-05
Human toxicity, cancer [kg 1,4-DB eq.]	2.73E-03	1.56E-02	5.62E-03	8.72E-03	4.35E-01	4.78E-02	3.25E-02	4.00E-02	5.34E-03
Human toxicity, non-cancer [kg 1,4-DB eq.]	1.84E-02	9.11E-02	1.66E-01	1.11E-01	1.72E-01	1.86E-01	1.10E-01	2.42E-01	6.12E-02
Ionizing Radiation [Bq C-60 eq. to air]	2.41E-01	1.31E-02	5.66E-03	2.58E-02	1.66E-02	2.02E-02	1.64E-02	1.88E-02	4.90E-03
Land use [Annual crop eq.·y]	1.55E-03	2.24E-01	7.99E-02	-3.58E-03	1.20E-01	1.27E-01	1.00E-01	1.52E-01	1.01E-01
Marine ecotoxicity [kg 1,4-DB eq.]	4.62E-04	2.88E-03	1.58E-03	6.60E-04	7.53E-03	3.55E-03	2.24E-03	2.84E-03	5.95E-04
Marine Eutrophication [kg N eq.]	2.56E-05	1.43E-04	1.45E-04	-5.42E-06	4.28E-04	4.76E-04	2.80E-04	1.98E-04	5.50E-05
Metal depletion [kg Cu eq.]	4.73E-05	2.50E-04	-4.02E-04	-2.27E-04	6.13E-03	1.64E-03	8.58E-04	5.60E-04	-1.22E-05
Photochemical Ozone Formation, Ecosystems [kg NOx eq.]	1.64E-04	7.15E-04	3.43E-04	1.89E-04	7.99E-04	1.15E-03	7.04E-04	9.68E-04	2.77E-04
Photochemical Ozone Formation, Human Health [kg NOx eq.]	1.54E-04	6.92E-04	3.35E-04	1.76E-04	7.30E-04	1.08E-03	6.37E-04	8.92E-04	2.69E-04
Stratospheric Ozone Depletion [kg CFC-11 eq.]	1.51E-08	1.02E-07	4.49E-07	-8.26E-08	6.16E-07	6.38E-07	4.67E-07	5.35E-07	2.81E-07
Terrestrial Acidification [kg SO2 eq.]	1.45E-04	5.10E-04	6.52E-04	2.99E-04	1.26E-03	1.47E-03	9.85E-04	1.14E-03	3.71E-04
Terrestrial ecotoxicity [kg 1,4-DB eq.]	4.72E-02	1.59E+00	5.28E-01	2.78E-01	3.30E+00	1.12E+00	8.47E-01	1.52E+00	4.85E-01
Damage to human health [DALY]	2.12E-07	5.00E-07	5.67E-07	7.54E-07	2.41E-06	1.25E-06	8.56E-07	9.52E-07	1.79E-07
Damage to ecosystems [species.yr]	5.76E-10	3.26E-09	2.08E-09	2.02E-09	3.85E-09	4.46E-09	3.19E-09	3.66E-09	1.32E-09
Damage to resource availability [$]	2.16E-02	9.82E-03	1.06E-02	3.09E-02	2.75E-02	3.07E-02	2.70E-02	2.76E-02	4.04E-03

* (R):Reusable

Table S11. Consumers' preference survey Questions.

A/A	Question
	How many times do you use drinking straws in your daily life? (Please specify the type)
	How many plastic straws do you estimate you use per year? (before the ban and after the ban, please specify)
	How comfortable did you feel using traditional polypropylene (PP) straws? (Scale: 1-5) (before their ban)
	Do you believe PP straws have a high climate impact? (Yes/No)
	How appropriate do you find paper straws for daily use? (Scale: 1-5)
	Do you consider paper straws to be cost-effective? (Yes/No)
	Would you consider using reusable straws as an alternative to PP straws? (Yes/No)
	How willing are you to use silicone straws? (Scale: 1-5)
	How willing are you to use metallic straws? (Scale: 1-5)
	How willing are you to use glass straws? (Scale: 1-5)
	How willing are you to use bamboo straws? (Scale: 1-5)
	How willing are you to use wheat straws? (Scale: 1-5)
	How willing are you to use Plastic (PLA, PHA) straws? (Scale: 1-5)
	Do you believe the ban on PP straws is due to their non-recyclability? (Yes/No)
	Do you think the mass and collection routes of PP straws hinder recycling with current techniques? (Yes/No)
